# A Spontaneous *Fatp4/Scl27a4* Splice Site Mutation in a New Murine Model for Congenital Ichthyosis

**DOI:** 10.1371/journal.pone.0050634

**Published:** 2012-11-30

**Authors:** Jianning Tao, Maranke I. Koster, Wilbur Harrison, Jennifer L. Moran, David R. Beier, Dennis R. Roop, Paul A. Overbeek

**Affiliations:** 1 Department of Molecular and Cellular Biology, Baylor College of Medicine, Houston, Texas, United States of America; 2 Department of Molecular and Human Genetics, Baylor College of Medicine, Houston, Texas, United States of America; 3 Department of Dermatology and Charles C. Gates Center for Regenerative Medicine and Stem Cell Biology, University of Colorado Anschutz Medical Campus, Aurora, Colorado, United States of America; 4 Division of Genetics, Department of Medicine, Brigham and Women’s Hospital, Harvard Medical School, Boston, Massachusetts, United States of America; CNRS UMR7275, France

## Abstract

Congenital ichthyoses are life-threatening conditions in humans. We describe here the identification and molecular characterization of a novel recessive mutation in mice that results in newborn lethality with severe congenital lamellar ichthyosis. Mutant newborns have a taut, shiny, non-expandable epidermis that resembles cornified manifestations of autosomal-recessive congenital ichthyosis in humans. The skin is stretched so tightly that the newborn mice are immobilized. The genetic defect was mapped to a region near the proximal end of chromosome 2 by SNP analysis, suggesting *Fatp4*/*Slc27a4* as a candidate gene. *FATP4* mutations in humans cause ichthyosis prematurity syndrome (IPS), and mutations of *Fatp4* in mice have previously been found to cause a phenotype that resembles human congenital ichthyoses. Characterization of the *Fatp4* cDNA revealed a fusion of exon 8 to exon 10, with deletion of exon 9. Genomic sequencing identified an A to T mutation in the splice donor sequence at the 3′-end of exon 9. Loss of exon 9 results in a frame shift mutation upstream from the conserved very long-chain acyl-CoA synthase (VLACS) domain. Histological studies revealed that the mutant mice have defects in keratinocyte differentiation, along with hyperproliferation of the stratum basale of the epidermis, a hyperkeratotic stratum corneum, and reduced numbers of secondary hair follicles. Since Fatp4 protein is present primarily at the stratum granulosum and the stratum spinosum, the hyperproliferation and the alterations in hair follicle induction suggest that very long chain fatty acids, in addition to being required for normal cornification, may influence signals from the stratum corneum to the basal cells that help to orchestrate normal skin differentiation.

## Introduction

Keratinocytes in the mammalian epidermis are stratified into four cellular layers: stratum basale (basal), stratum spinosum (spinous), stratum granulosum (granular), and stratum corneum (cornified). The basal cells are proliferative and express characteristic markers, including keratins 5 and 14. The spinous cells have withdrawn from the cell cycle and express keratins 1 and 10. The granular cells synthesize lamellar bodies/keratohyalin granules, and then convert to corneocytes, which are enucleated and encapsulated by a modified plasma membrane termed the corneocyte envelope (CE). The CE protects against water loss (an inside-outside barrier) and against insults such as microbes from without (an outside-inside barrier) [1], [2]. The lipid matrix of the CE contains ceramides, long chain fatty acids, and cholesterol and its esters, which are deposited from the lamellar bodies of the granular cells. During epidermal development in mammals, defects in the production of structural proteins, or enzymes, or lipid components of the CE result in barrier defects and/or congenital ichthyoses [2], [3].

Mammalian very-long-chain acyl-CoA synthetases (ACSVLs) or fatty acid transport proteins (FATPs) are a family of six related proteins [4]. These proteins contain two “signature” domains: the ATP/AMP domain which is required for ATP binding, and the VLACS/FATP domain (approximately 50 amino acids), which is required for fatty acid binding and enzymatic activity [5], [6]. The FATP genes have different expression patterns, and the proteins have different sub-cellular locations and substrate specificities. Defective ACSVLs/FATPs have been implicated in human diseases such as heart failure, obesity, diabetes/insulin resistance, cold intolerance, and fat mal-absorption [4], [7]. Furthermore, the most widely expressed member of this family is Fatp4, which is encoded by *Slc27a4* (solute carrier family 27 member 4 gene), and its broad expression pattern is suggestive of functions in many organs [8], [9]. In mammalian skin, Fatp4 protein is localized to the stratum granulosum and the stratum spinosum [9]–[11].

The physiological role of Fatp4 has been studied using mouse models. A retrotransposon insertion into exon 3 of *Fatp4* was identified in an autosomal recessive mouse mutant termed wrinkle-free (*wrfr*) [12]. Independently, a targeted knock-out of *Fatp4* (that affects exon 3) was generated and characterized [10]. In both cases, mutant mice are born with tight, thick, shiny skin and a defective skin barrier [10], [12]. The mutant mice die shortly after birth. In a third mouse model, deletion of *Fatp4* exons 2 and 3 was found to result in embryonic lethality prior to embryonic day 9.5 [13]. The reason for this discrepancy remains unknown. *Fatp4* has also been conditionally deleted in the adult mice [9]. By gross appearance these mice appear normal, but mild histological abnormalities are present in the epidermis, supporting a role for Fatp4 in skin homeostasis [9]. Using a transgenic approach, expression of Fatp4 in suprabasal keratinocytes was found to be sufficient to rescue the *wrfr* mutant phenotype, resulting in viable and fertile mice [8]. The *Fatp4* mutant mice were initiatively suggested to be a mouse model for a very rare human genetic disorder, lethal restrictive dermopathy [1], [10], [12], [14]. Restrictive dermopathy in humans has now been linked to mutations in the zinc metalloproteinase ZMPSTE24 whereas mutations in Fatp4 in humans cause ichthyosis prematurity syndrome (IPS) [11], [15]. IPS is a rare disorder of cornification classified as one of the autosomal-recessive congenital ichthyoses [16]. Key features in IPS are complications resulting from prematurity born with thick caseous desquamating epidermis, typically showing lipid membrane packages in the granular and cornified cells, then a lifelong nonscaly ichthyosis with dermal atopic dermatitis-like inflammation and severe itching [17].

**Figure 1:**
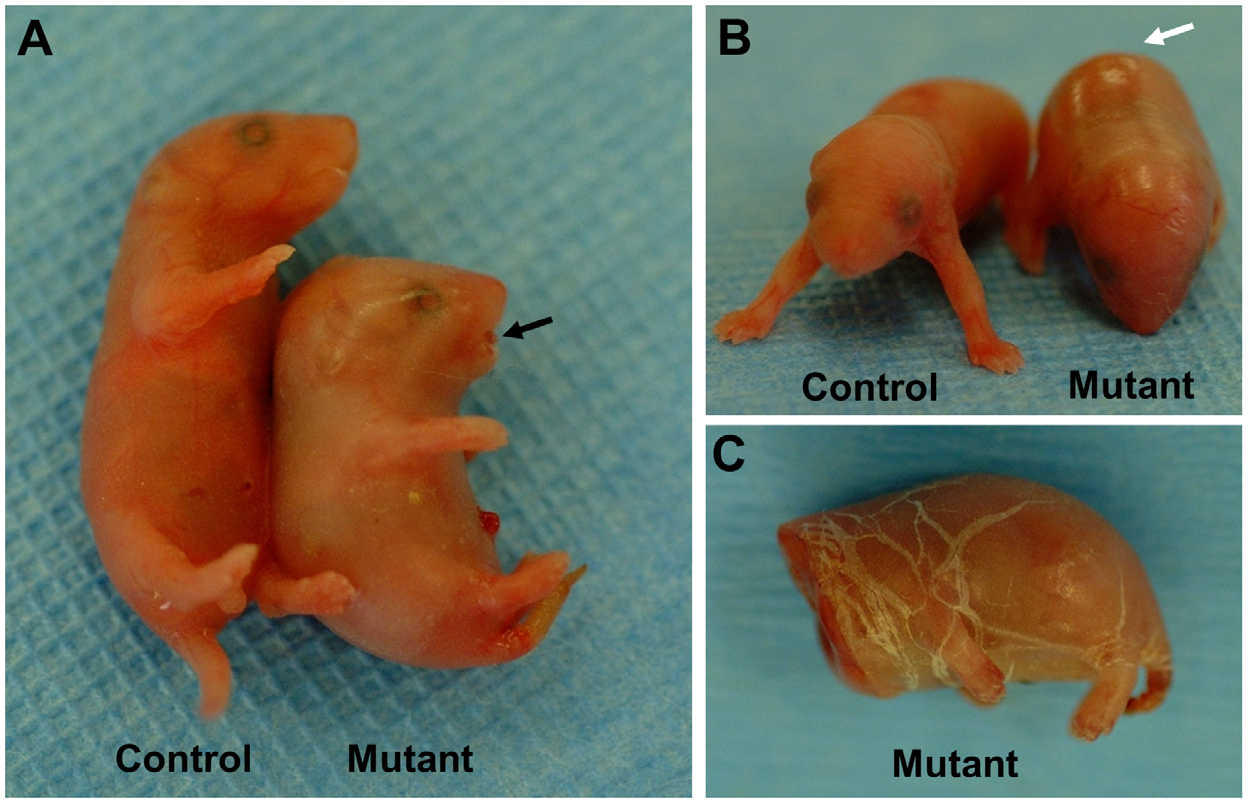
Newborn phenotype. A,B. Mutant newborn mice exhibited a protruding tongue (black arrow in A) and taut, smooth, shiny skin (white arrow in B). The skin was so tight that the newborn mice were unable to extend their limbs or to straighten their torso. Physical stretching of the skin (e.g. during decapitation) caused the skin to crack a multiple sites (C), resembling the phenotype of congenital ichthyosis in humans.

In the current study, we describe the identification and characterization of a spontaneous mutation in mouse *Fatp4* that results in autosomal recessive congenital ichthyosis. At birth, the mutant mice have smooth hyperkeratotic skin that is stretched so tightly that they are unable to extend their limbs or to straighten their torso. Histological studies revealed defects in epidermal differentiation and cornification. The mutation was mapped to chromosome 2, band A3/B, by SNP analysis, thus suggesting *Fatp4/Slc27a4* as a candidate gene. Sequencing studies revealed a spontaneous mutation in the splice donor sequence at the 3′-end of exon 9, resulting in exon skipping, a shift in reading frame, and the presence of a premature stop codon. The mutation results in loss of the C-terminal 243 amino acids of Fatp4, including the VLACS domain. The *Fatp4* mutant mice exhibit alterations in the stratum corneum that are similar to the defects seen in IPS [11], presumably reflecting a role for very long chain fatty acids in the formation and function of lamellar bodies. The *Fatp4* mutants also show basal cell hyperproliferation and a reduction in secondary hair follicle induction, suggesting the possibility that very long chain fatty acids synthesized in the superficial epidermis may, directly or indirectly, help to establish the proper prenatal balance between proliferation and differentiation of the basal cells.

**Figure 2:**
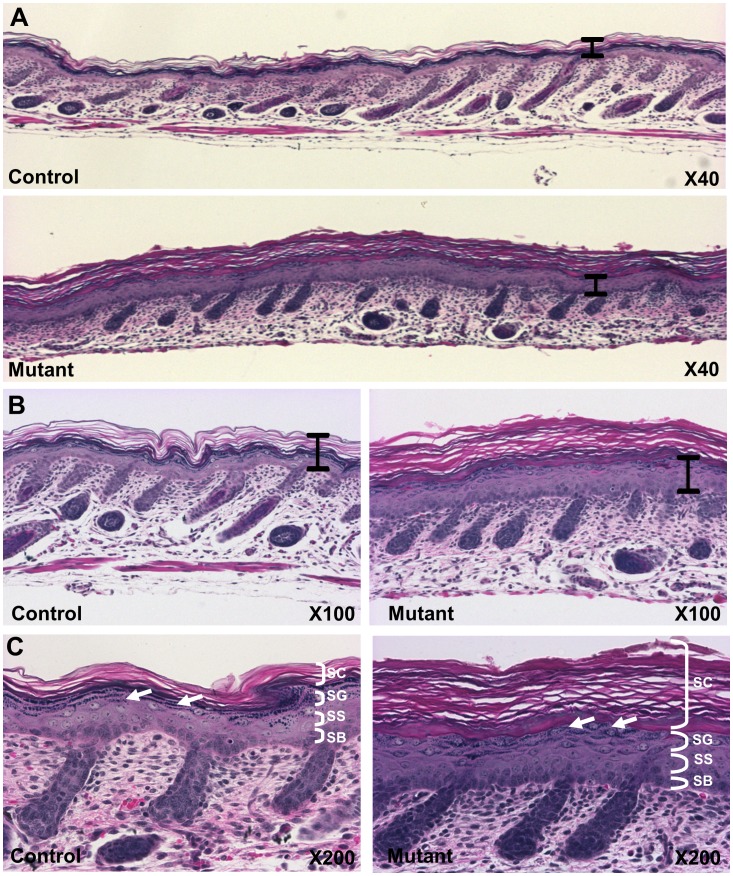
Altered cornification and epidermal differentiation. A,B,C. Dorsal skin was sectioned and stained with hematoxylin and eosin. Mutant epidermis was notably thicker and hyperkeratotic compared to the control (A, B, black bars are identical lengths.), and had significantly fewer hair follicles. Higher magnifications (B,C) show thicker stratum corneum (SC) and changes in keratohyalin granules in the stratum granulosum (SG) (white arrows). Abbreviations: stratum basale (SB), stratum spinosum (SS).

**Figure 3:**
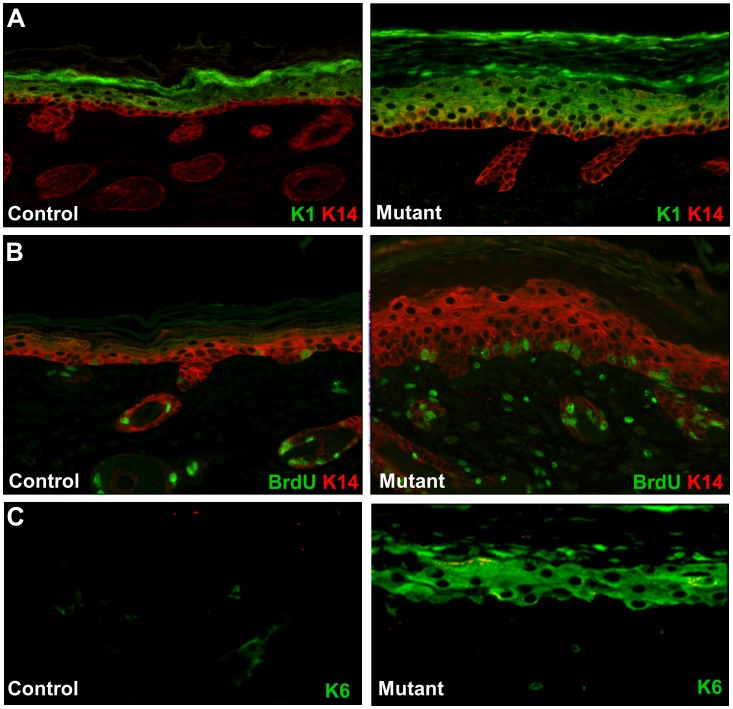
Hyperproliferation and altered expression of keratin markers. A. Immunostaining with anti-K14 (red) and anti-K1 (green) antibodies. K14 expression in the control epidermis is predominantly in the stratum basale of the epidermis (left panel). In the mutant epidermis, K14 was detected in both basal and suprabasal layers (red and yellow color in right panel and panel B). Suprabasal differentiation marker K1 was detected in suprabasal cells of both mutant and control skin. The suprabasal layer in the mutant was thicker than the control. B. BrdU incorporation (green) and keratin K14 expression (red) were visualized by immunostaining. The mutant epidermis showed more than twice as many BrdU-staining cells in the basal epithelium. C. Immunostaining for K6 (green). K6 is not detected in the control epidermis (left panel), but K6 is strongly expressed in the suprabasal cells in the mutant. Original magnifications: X200.

## Materials and Methods

### SNP Mapping, RT-PCR, and Sequencing

Genomic DNA from mouse tails was isolated [18] and a custom Illumina Golden Gate whole genome SNP panel was used for mapping essentially as described in Moran et al. [19]. Total RNA from wild-type and mutant dorsal skin was extracted with the RNeasy Mini Kit (Qiagen). First-strand cDNA was synthesized using the Superscript cDNA first strand synthesis kit (Invitrogen). Segments of *Slc27a4* cDNA were amplified by PCR using the following pairs of primers: exon1(sense, S) 5′-GAGGTGCACGGACTCAGAAG and exon3(antisense, AS) 5′-GAAGGTCCAGTGAGTGTCTGTG; exon3 (S) 5′-CTGTTTG CTTCAATGGTACAGC and exon6 (AS) 5′-CCAGGGAAGCCATACGATAATA; exon4 (S) 5′-ACCCAGACAAGGGTTTTACAGA and exon9 (AS) 5′-TTGACACGTACC AAACGGATAG; exon8 (S) 5′-GGCCACTGAATGCAACTGTAG and exon11 (AS) 5′-CAACACCATAAACTGCCACATC; exon11 (S) 5′-GAGCTGGGTTACCTGTACTTCC and exon13 (AS) 5′-CTAGGGCTCTGAATCCAGCAT. Primers exon 8 (S) and exon 11 (AS) amplified a smaller band from mutant cDNA compared to wild-type cDNA. Both bands were sequenced. For PCR amplification of the genomic region encompassing exon 9 of *Slc27a4*, we used the following primers: 5′-CCACTGAATGCAACTGTAGCC (exon 8, sense) and 5′-TAAAGCAGAACCCACACTCAGA (intron 9, antisense). A 435 bp fragment was amplified and sequenced.

**Figure 4:**
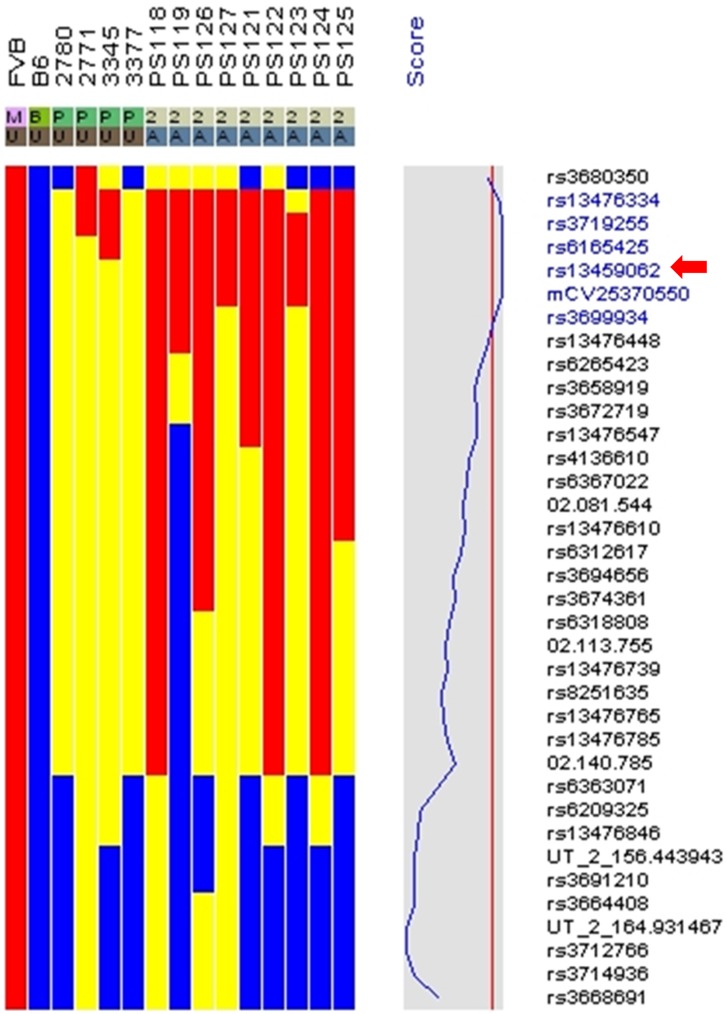
SNP mapping. Genomic DNA was analyzed from 4 heterozygous parents (2780, 2771, 3345 and 3377), and from 9 mutant offspring (PS118, 119, 121–127). Since the mutation occurred on an FVB background, the affected mice should be homozygous for FVB alleles (red color) that are linked to the mutation. Homozygosity for the C57BL/6 allele (B6) is indicated in blue. Carriers of both alleles are indicated in yellow. The critical region is centered around the SNP named rs13459062 (red arrow). This sequence is located near 30 MB on mouse chromosome 2. P, parental; A, affected; U, unaffected.

**Figure 5:**
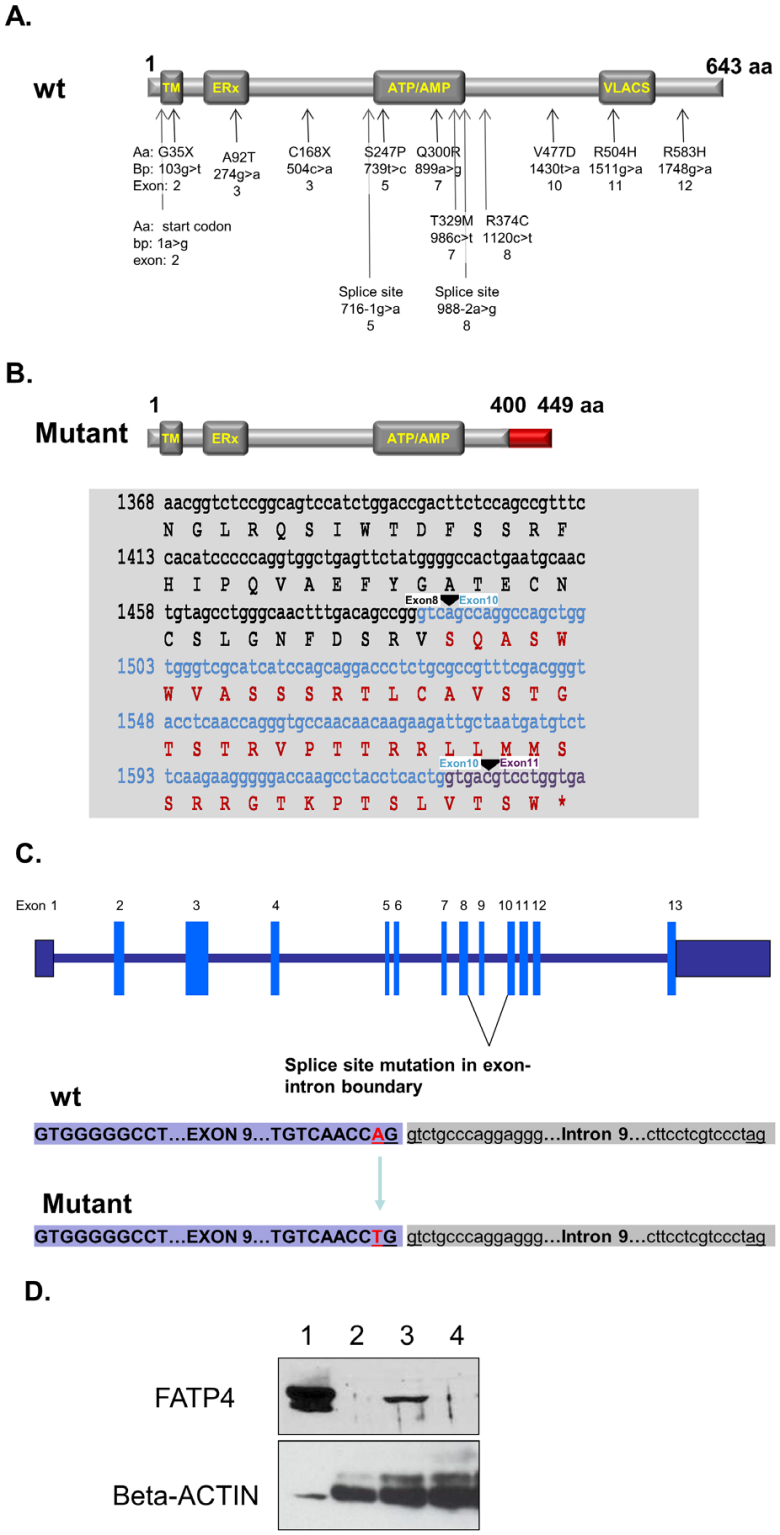
Mutations in Slc27a4/Fatp4. A. Schematic drawing of the FATP4 protein and a summary of the mutations found in human IPS patients. Functional domains include the N-terminal transmembrane region (TM), the ER localization signal (ERx; aa 47–102), the ATP/AMP motif involved in ATP binding (ATP/AMP; aa 243–345) and the conserved VLACS/FATP motif of importance for fatty acid binding (FATP; aa 500–551). Arrows indicate the positions of published mutations [11], [28]–[31]. Aa, amino acids and their position in the protein; bp, nucleotide changes and their location relative to the start codon; the exon name is listed last for each mutation. B. Pigskin Fatp4 protein, cDNA sequencing results and the encoded amino acid sequence. The mutant protein is predicted to be 449 aa in length, with the last 49 aa (in red) encoded after a frameshift caused by the loss of exon 9. The truncated Fatp4 protein is missing the FATP/VLACS domain. Exon 8 nucleotides and wt amino acids are in black. Exon 10 nucleotides are in light blue and exon 11 nucleotides are in purple. The black triangles indicate exonic boundaries. C. Schematic drawing of altered splicing plus the genomic sequencing results. The pigskin mutants have a point mutation (A to T) in the splice donor site for Slc27a4 exon 9. D. Lack of full-length Fatp4 protein in the skin of pigskin mutant mice. A Western blot analysis was performed using an anti-Fatp4 antibody generated against a C-terminal peptide antigen. Lane 1 is a positive control (10 ug cell lysate from 293T cells transfected with mouse Fatp4 cDNA (NM_011989)). Lane 2: 25 ug whole skin lysate from a pigskin newborn. Lane 3: 50 ug whole skin lysate from a normal littermate. Lane 4: 50 ug whole skin lysate from a pigskin newborn. Beta-actin was used as a loading control.

**Figure 6:**
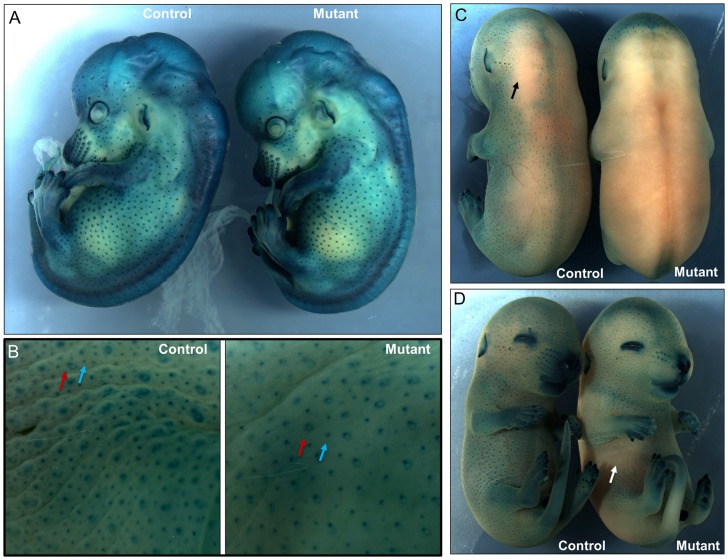
Alteration of skin structure at the earlier stages of pigskin mutants. Hair follicle induction was assayed using a BMP4-lacZ reporter line from E14.5 to E16.5. Embryos were incubated in solution with 5-bromo-4-chloro-3-indolyl-b- D-galactoside (X-gal). X-gal cleavage by beta-galactosidase results in dark blue staining. A. At E14.5, representative control and pigskin mutant embryos display blue-stained primary hair follicles (PHFs) and vibrissal follicles. There were similar numbers of PHFs in the lateral side of control and mutant mice. B. Peeled skin from representative control and pigskin E16.5 embryos was stained with X-gal. Primary hair follicles (PHFs) are larger and often show an unstained core and a distinctive ring shape (red arrows). Secondary hair follicles (SHFs) are smaller and are more numerous. The ratio of SHFs to PHFs in the mutant epidermis is significantly decreased compared to that of the control (n = 3, p<0.01, see materials and methods). **C, D.** Intact E15.5 control embryos display blue-stained hair follicles over most of their surface, except for local regions on the dorsum with limited staining (black arrow) (C). In contrast, pigskin mutant embryos had large portions of their back and lateral skin as well as ventral sites (white arrow) that were not stained by X-gal, indicating alternated permeability at E15.5 (D).

### Histological Analysis and Immunofluorescence

Skin from newborn mice was fixed in 10% neutral buffered formalin (NBF), and embedded in paraffin. Sections were cut at 5 um and stained with hematoxylin/eosin. For *in vivo* BrdU incorporation, newborns were injected i.p. with 250 µg/g BrdU (Sigma) in 0.9% sterile saline as described [20]. After 1 h, tissue was fixed in 10% NBF. Primary antibodies used for immunofluorescence were FITC anti-BrdU (Becton Dickinson, Franklin Lakes, NJ), guinea pig anti-K14 [21], rabbit anti-K1 [21], and rabbit anti-K6 [22]. Secondary antibody conjugates used were Alexa594-conjugated goat anti-guinea pig and 488-conjugated goat anti-rabbit (Molecular Probes). All experiments involving mice were approved by the Baylor IACUC.

### Western Blot Analysis

Newborn dorsal skin was homogenized and extracted in lysis buffer (10 mM Tris-Cl at pH 7.4, 5 mM EDTA, 100 mM NaCl, 1% Triton X-100 with Complete Proteinase Inhibitors Cocktail) (from Roche). 293T cells (human kidney cells) were grown in supplemented DMEM medium (Invitrogen), and transfected with an expression construct encoding mouse *Fatp4* (NM_011989) (purchased from Open Biosystems) using FuGene6 (Roche). Cells were harvested for Western blot analysis 48 h after transfection. The primary antibody (1∶500) was a rabbit antibody generated against the C-terminal 35 amino acids of mouse Fatp4, a gift from Dr. Paul A. Watkins (Kennedy Krieger Institute) [23]. After incubation with an HRP-conjugated anti-rabbit secondary antibody, protein bands were visualized using Super Signal West Pico Substrate (Pierce). An antibody against beta-actin (Sigma-Aldrich, cat#: A2228) was used as a loading control.

### X-gal Staining of Whole Embryos and Skin

To assess the pattern of hair follicle induction, we used a BMP4-lacZ reporter line [24] and we assayed for ß-galactosidase activity by X-gal staining as described previously [25]. Briefly, males that were compound heterozygous for the *Fatp4* mutation and for BMP4-lacZ, were mated to females heterozygous for the *Fatp4* mutation. Embryos were genotyped by PCR using one pair of primers to amplify the wild type allele (Ex8 (S), 5′-CCACTGAATG CAACTGTAGCC-3′ and Ex9(WT,AS), 5′-TCCATTCCCTCCTGGGCAGACCT-3′ and a different antisense primer (Ex9, pigskin AS, 5′-TCCATTCCCTCCTGGGCAGACCA-3′ to assay for the mutant allele. Amplification bands were 360 bp. Mouse embryos or peeled skin were harvested from timed pregnancies and fixed in 2% paraformaldehyde plus 0.2% glutaraldehyde in 0.1 M phosphate buffer (pH 7.3) at 4°C for 1 hour. Embryos or skin were rinsed three times (30 minute each) in washing solution containing 0.1 M phosphate buffer (pH 7.3), 2 mM MgCl2, 0.01% sodium deoxycholate, and 0.02% NP-40. Embryos were then stained at 4°C for 12 hours in X-gal staining solution (washing solution plus 5 mM potassium ferrocyanide, 5 mM potassium ferricyanide, and 1 mg/mL X-gal). Stained embryos or skin were rinsed in phosphate-buffered saline (PBS; pH 7.4) and stored in 70% ethanol. After staining, embryos were photographed using a 35 mm Nikon digital camera and images were processed with Adobe Photoshop. All of blue hair follicles in the lateral body (1 mm x 1 mm area) of E14.5 embryos were counted (at least three embryos in each genotype). A strong-stained blue dot with an unstained core and a distinctive ring shape from the skin of E16.5 embryos was counted as primary hair follicles (PHFs) while other smaller stained blue dots were counted as secondary hair follicles (SHFs). Statistical significance (p values) was computed by using Student’s t test. A p value of less than 0.05 was considered statistically significant. Image J software was used to count hair follicles [26].

## Results

### New Mouse Mutant with Autosomal Recessive Congenital Ichthyosis

Within a breeding colony of FVB mice, a few of the offspring from one breeder pair exhibited an abnormal skin phenotype at birth. Some pups were born with tight, smooth, shiny skin (Fig. 1A and B). The skin was stretched so tightly that the newborns were immobilized in a fetal position, unable to extend their body or their limbs. The characteristic appearance of the skin led us to describe the newborns as “pigskin” mutants. The mutant mice had a small jaw and protruding tongue (Fig. 1A). In some mutants, the toes and the tip of the tail showed signs of necrosis at birth (not shown). Although some of the mutants were able to breathe, they died shortly (within a few hours) after birth. We found no milk in their stomachs, indicating they were unable to suckle. Stretching of the skin caused widespread cracking (Fig. 1C), reminiscent of congenital ichthyoses in humans [11], [27]. Breeding studies confirmed that the pigskin phenotype was inherited as an autosomal recessive trait.

### Aberrant Epidermal Differentiation and Hyperkeratosis

Skin from newborn mice was harvested and processed for histological analyses. The exterior surface of the skin and the epidermal-dermal junction were flattened compared with normal skin (Fig. 2A). The mutant epidermis was notably thicker than normal. The stratum corneum of the mutant epidermis was considerably thicker than control epidermis (Fig. 2B and 2C), indicative massive hyperkeratosis (abnormal accumulation of cornified cells). The cells of the stratum granulosum showed changes in the patterning, size, and distribution of the dense basophilic keratohyalin granules in the mutant skin (Fig. 2C, white arrows). These granules contain aggregated keratin fibers and lipids, which help to build the epidermal barrier. The stratum spinosum was characterized by an increased number of cell layers in the mutant. No significant abnormalities were identified in other tissues (data not shown).

To investigate proliferation and differentiation of the keratinocytes, we employed keratin immunostaining and BrdU incorporation assays (Fig. 3). In control skin, Keratin K14 expression is detected in the basal epithelial cells while keratin K1 reactivity was observed in all suprabasal cell layers (Fig. 3A). The mutant epidermis showed K14 labeling in more suprabasal layers (Fig 3A and 3B). BrdU-labeled cells were detected sporadically in the stratum basale in control epidermis, but more than twice as many BrdU-labeled cells were found in the mutant epidermis (Fig. 3B). We also assayed the epidermis for expression of Keratin K6, a marker of aberrant epidermal differentiation. K6-labeled cells were strongly detected in the suprabasal layers of the mutant epidermis, but not in the control epidermis (Fig. 3C). These findings indicate that all layers of the skin are affected in the pigskin mutant.

### SNP Mapping of the Pigskin Mutation

The pigskin mutation arose on an FVB background. In order to map the mutation, we mated pigskin carrier males to C57BL/6J partners. The F1 offsprings were used for test matings to identify mice that carried the pigskin mutation. Carriers were mated to each other, and the F2 offspring were again mated to identify carriers of the pigskin mutation. F2 carriers and their mutant offspring were used for SNP analysis [19]. We analyzed genomic DNA from four carrier parents, and nine affected newborns (Fig. 4) as well as the parental FVB and C57 lines. SNP mapping identified a candidate region of the genome centered around rs13459062, which is located near 30 Mb on mouse chromosome 2.

Since *Fatp4* (*Slc27a4*) maps at 29.5 Mb on chromosome 2, it lies within the critical region of the genome. Mouse *Fatp4* and human *FATP4* encode homologous proteins. Recent studies have identified mutations in *FATP4* in human patients with IPS [11], [28]–[31] and the mutations found so far are point mutations (summarized in Figure 5A). In order to look for defects in the *Fatp4* coding sequences in the pigskin mutants, segments of the mRNA were amplified by RT-PCR from newborn mutant and wild-type skin (see Materials and Methods). Using an exon 4 sense primer and an antisense primer from exon 9, wild-type skin gave an amplification band, while the mutant skin did not (data not shown). Using a sense primer from exon 8 and an antisense primer from exon 11, the mutant RNA gave an amplification band that was about 120 bp smaller than wild-type (data not shown). Sequencing revealed that exon 9 was completely missing from the mutant transcript (Fig. 5B). The loss of exon 9 (127 bp) causes a shift in the coding frame so the pigskin transcript encodes a truncated protein with only 449 amino acids. Of these, only the first 400 amino acids are from wild-type Fatp4 (see Fig. 5B). The truncated protein will be missing the conserved VLACS/FATP domain. Based on the RT-PCR results, we designed primers to amplify exon 9 and the flanking genomic sequences by direct PCR from genomic DNA. Sequencing of the amplified bands revealed a point mutation (an A to T transversion) in the consensus splice donor sequence at the 3′-end of exon 9 in the mutant genome (Fig. 5C). Since antibodies against the N-terminus of Fatp4 are not available, westerns were performed using a Fatp4 antibody generated against a peptide from the C-terminus of Fatp4 (Fig. 5D). No band was detected in extracts from mutant skin (Fig. 5D, lanes 2 and 4), verifying the prediction that the full length Fatp4 protein is not synthesized by the pigskin mutants. Together, we conclude that this point mutation in *Slc27a4* is the cause of the pigskin phenotype.

### Alternated Hair Follicle Growth and Skin Structure at the Earlier Stages of Pigskin Embryos

The shiny and smooth phenotype of the newborn pigskin mutant skin and our histological studies suggested that there might be a defect in hair follicle induction, consistent with the previous report that the *wrfr* mutant mice had impaired hair development with fewer developing hair follicles. In mice, tylotrich or primary hair follicles (PHFs) are induced beginning at E14 and are characterized by a large hair bulb and two sebaceous glands [32]–[34]. Nontylotrich or secondary hair follicles (SHFs) begin to differentiate at approximately E16 [33]. In order to assess whether PHFs or SHFs or both are affected in the mutant mice, we intercrossed pigskin mice with a BMP4-lacZ reporter strain (from Dr. Yas Fututa) [24]. These mice have a lacZ reporter inserted into the endogenous BMP4 locus by homologous recombination [24]. BMP4 expression is robust at the onset of primary and secondary hair follicle induction [35], [36]. Interestingly, at E14.5, the pigskin mutants showed a similar pattern, distribution and density of PHFs compared to the control (Fig. 6A). There were no significant changes of PHFs numbers in the control and mutant mice (the numbers per square millimeter: wt  = 23.5±2.1, pigskin  = 24.6±3.2, n = 3, *p* = 0.57). However, at E16.5, skin from the pigskin mutants showed a significant decrease in the numbers of hair follicles compared to the control littermates (Fig. 6B). Notably, the ratio of SHFs to PHFs in the mutant epidermis is significantly decreased (the ratio of SHFs to PHFs: wt  = 3.01±0.20, pigskin  = 1.27±0.28, n = 3, *p*<0.01). Thus, the *Fatp4* mutation leads to a reduction of number of SHFs.

Skin barrier formation follows a precise spatiotemporal pattern during embryogenesis [37]. The timing of development of the epidermal barrier can be examined by incubation of embryos in X-Gal solution at low pH. Detection endogenous beta-galactosidase activity occurs only prior to development of the barrier. Using this approach, previous studies demonstrated that Fatp4 mutant skin has a barrier defect at E18.5 and in newborn mice [9], [10], [12]. In our study, when we performed X-gal staining to detect the BMP4-LacZ activity in hair follicles, we found that X-gal penetrated the epidermis and stained the hair follicles in both control and mutant embryos at E14.5 (Fig 6.A). However, interestingly, at E15.5, blue staining was almost completely absent from the dorsal skin of the mutant embryos, but was observed throughout the dorsum of the control littermates (Fig. 6C). Blue staining was observed in the ventral follicles in the control embryos, but was partially lost on the ventral side of the mutants (Fig. 6D, arrow). At E16.5, both control and mutant embryos had no X-gal staining of intact dorsal and lateral skin (data not shown). Together, these data suggest that skin barrier development has been affected by E15.5 in the mutant embryos.

## Discussion

We have identified and characterized a new mouse model for autosomal recessive, non-bullous, congenital ichthyosis. Mutant mice are born with a “tight skin” phenotype. The skin is stretched so tightly that the mice are unable to move their limbs. The mutant mice have a distinctive protruding tongue (Fig. 1A), are unable to suckle, and die shortly after birth. Histological analysis of the skin showed hyperkeratosis (Fig. 2A–C), defects in the lamellar bodies of the granular cells (Fig. 2B,C), hyperproliferation (Fig. 3B) and altered of keratin marker gene expression (Fig. 3A–C). The mutation was mapped using SNP analysis (Fig. 4). Molecular characterization of the Fatp4/Slc27a4 transcript (Fig. 5B) and gene (Fig. 5C) identified a point mutation in the splice donor sequence of exon 9, resulting in exon skipping during processing of the primary transcript. The altered transcript encodes a predicted truncated Fatp4 protein that lacks the conserved VLACS domain (Fig. 5C). Western blots confirmed the loss of expression of the full-length protein in newborn skin (Fig. 5D). This spontaneous mutation provides further evidence that Fatp4 is essential for proper cornification and barrier formation in the epidermis.

Currently, autosomal recessive congenital ichthyosis (ARCI) in humans is associated with mutations in genes including *TGM1* (MIM*190195), *ABCA12* (MIM *607800), *ALOXE3* (MIM *607206), *ALOX12B* (MIM*603741), *ABHD5* (MIM *604780), *NIPAL4* (MIM *609383), *CYP4F22* (MIM*611495), and *SLC27A4/Fatp4* (MIM *604194) related to this study [38]. Among them, mutations in *ABCA12*, a member of the ABC transporter superfamily, cause Harlequin ichthyosis (HI), a disorder that presents at birth with a thick, tight skin that is susceptible to cracking [1]. In keratinocytes, ABCA12 is thought to regulate the transfer of glucosyl-ceramides into lamellar bodies. Loss of ABCA12 function in mice causes hyperkeratosis (expanded stratum corneum) and malformed lamellar bodies [39]. These phenotypes resemble the “pigskin” phenotype in the *Fatp4* mutant mice [10], [12]. Like ABCA12, Fatp4 plays an essential role in the construction or function of lamellar bodies [10]. Fatp4 functions as an acyl-CoA synthetase with specificity towards very long chain fatty acids including arachidonate (C20∶4) and lignocerate (C24∶0), which are essential for lamellar bodies [4]. We therefore predict that Fatp4 and ABC12 may cooperate either directly or indirectly in lamellar bodies to help produce the normal cornified envelope (CE). Mutations of TGM1 (keratinocyte transglutaminase 1), a calcium dependent enzyme that functions in cross-linking of epidermal structural proteins and lipids into the CE, cause lamellar ichthyosis [40]. Studies have showed that expression of TGM1 is directly regulated through its promoter by GRHL3/GET1, an epidermal-specific transcription factor [41], [42]. It will be interesting to know whether GRHL3/GET1 also regulates Fatp4 expression during epidermal development. In the future, determination of the upstream regulators of Fatp4 expression and its interaction with other proteins mutated in ARCI may give us insights into the molecular events that specify the unique architecture of the CE.

Recently, human mutations of FATP4 have been found to cause IPS, a well-defined congenital ichthyosis subtype [11], [28]–[31]. Klar et al first reported FATP4 mutations in IPS patients from the Scandinavia, Middle East and North Africa [11]. IPS is more prevalent in Norway and Sweden with an estimated local carrier frequency of one in 50 suggesting a founder mutation. Outside of this region, only a few cases have been reported in other countries including Germany, Finland, Italy, Denmark and France. All patients from this region were found homozygous or compound heterozygous for p.C168X nonsense mutation. Up to now, thirteen distinct FATP4 mutations have been found in IPS patients (see summary in Figure 5A), including two nonsense mutations, eight missense mutations, one start site mutation, and two splice site mutations [11], [28]–[31]. Notably, all of patients with IPS were reported to present similar clinical features. In our case, a point mutation (an A to T transversion) in the consensus splice donor sequence at the 3′-end of exon 9 results in exon skipping and predicts synthesis of a truncated protein without the FATP/VLACS motif. The consensus genomic 5′ splice sequence in mammals, from −3 to +6 relative to the exon/intron boundary, is 5′-CAGGTAAGT. This sequence binds with perfect complementarity to the U1 snRNA. Although A is the nucleotide found most often at position −2 (64% of the time), T is present 14% of the time, so the presence of a T does not intrinsically prevent efficient splicing [43]–[45]. Looking specifically at intron 9 of mouse Slc27a4, the wild-type genomic sequence at the 5′-exon/intron boundary is 5′-CAGGTctGc. Six of these nine nucleotides match the consensus. In the pigskin mutant, the change of A to T at position −2 leaves only 5 nucleotides that match the consensus. Our findings imply that this change is sufficient to prevent effective use of this splice site.

The “pigskin” mutant mice display a comparable phenotype to the *wrfr* and Fatp4 knockout mice described in previous studies [10], [12]. However, the *wrfr* mutation was caused by a 230 bp retrotransposon insertion into Exon3 and the knockout mice were generated by deleting a genomic fragment containing exon3. Thus, the “pigskin” mice may be particularly useful to develop molecular therapies for IPS patients using targeted gene correction [46].

Since Fatp4 protein is detected specifically in suprabasal cells [10] and targeted expression in those cells is sufficient to rescue the mutant phenotype [8], we hypothesize that the basal cell hyperproliferation, the abnormal expression of K6, and the alterations in secondary hair follicle induction in Fatp4 mutants all reflect indirect, non-cell autonomous, responses to the loss of synthesis and release of very long chain fatty acid derivatives from the spinous and granular cells. We hypothesize that very long chain fatty acids synthesized by Fatp4 may provide both metabolic and regulatory functions that help to modulate epidermal homeostasis and differentiation.

In summary, we have identified a new mouse model for autosomal recessive congenital ichthyosis. The pigskin mutant mice, like most human patients with IPS, have a point mutation in the gene encoding Fatp4. These new mice provide a potential model system in which to study the feasibility of achieving gene therapy in the epidermis using homology-based strategies to correct single base mutations.

## References

[1] Elias PM, Williams ML, Holleran WM, Jiang YJ, Schmuth M (2008) Pathogenesis of permeability barrier abnormalities in the ichthyoses: inherited disorders of lipid metabolism. J Lipid Res 49: 697–714. 18245815 10.1194/jlr.R800002-JLR200 PMC2844331

[2] Proksch E, Brandner JM, Jensen JM (2008) The skin: an indispensable barrier. Exp Dermatol. 10.1111/j.1600-0625.2008.00786.x 19043850

[3] Pouillot A, Dayan N, Polla AS, Polla LL, Polla BS (2008) The stratum corneum: a double paradox. J Cosmet Dermatol 7: 143–148. 18482020 10.1111/j.1473-2165.2008.00379.x

[4] Watkins PA (2008) Very-long-chain acyl-CoA synthetases. J Biol Chem 283: 1773–1777. 18024425 10.1074/jbc.R700037200

[5] Zou Z, DiRusso CC, Ctrnacta V, Black PN (2002) Fatty acid transport in Saccharomyces cerevisiae. Directed mutagenesis of FAT1 distinguishes the biochemical activities associated with Fat1p. J Biol Chem 277: 31062–31071. 12052836 10.1074/jbc.M205034200

[6] DiRusso CC, Darwis D, Obermeyer T, Black PN (2008) Functional domains of the fatty acid transport proteins: studies using protein chimeras. Biochim Biophys Acta 1781: 135–143. 18258213 10.1016/j.bbalip.2008.01.002 PMC2274961

[7] Gimeno RE (2007) Fatty acid transport proteins. Curr Opin Lipidol 18: 271–276. 17495600 10.1097/MOL.0b013e3281338558

[8] Moulson CL, Lin MH, White JM, Newberry EP, Davidson NO, et al (2007) Keratinocyte-specific expression of fatty acid transport protein 4 rescues the wrinkle-free phenotype in Slc27a4/Fatp4 mutant mice. J Biol Chem 282: 15912–15920. 17401141 10.1074/jbc.M701779200

[9] Herrmann T, Grone HJ, Langbein L, Kaiser I, Gosch I, et al (2005) Disturbed epidermal structure in mice with temporally controlled fatp4 deficiency. J Invest Dermatol 125: 1228–1235. 16354193 10.1111/j.0022-202X.2005.23972.x

[10] Herrmann T, van der HF, Grone HJ, Stewart AF, Langbein L, et al (2003) Mice with targeted disruption of the fatty acid transport protein 4 (Fatp 4, Slc27a4) gene show features of lethal restrictive dermopathy. J Cell Biol 161: 1105–1115. 12821645 10.1083/jcb.200207080 PMC2173002

[11] Klar J, Schweiger M, Zimmerman R, Zechner R, Li H, et al (2009) Mutations in the fatty acid transport protein 4 gene cause the ichthyosis prematurity syndrome. Am J Hum Genet 85: 248–253. 19631310 10.1016/j.ajhg.2009.06.021 PMC2725242

[12] Moulson CL, Martin DR, Lugus JJ, Schaffer JE, Lind AC, et al (2003) Cloning of wrinkle-free, a previously uncharacterized mouse mutation, reveals crucial roles for fatty acid transport protein 4 in skin and hair development. Proc Natl Acad Sci U S A 100: 5274–5279. 12697906 10.1073/pnas.0431186100 PMC154335

[13] Gimeno RE, Hirsch DJ, Punreddy S, Sun Y, Ortegon AM, et al (2003) Targeted deletion of fatty acid transport protein-4 results in early embryonic lethality. J Biol Chem 278: 49512–49516. 14512415 10.1074/jbc.M309759200

[14] Armbrust S, Hoffmann R, Jochum F, Neumann LM, Fusch C (2005) Restrictive dermopathy associated with transposition of the great arteries and microcolon: a rare neonatal entity with new symptoms. Arch Dermatol 141: 611–613. 15897383 10.1001/archderm.141.5.611

[15] Navarro CL, Sandre-Giovannoli A, Bernard R, Boccaccio I, Boyer A, et al (2004) Lamin A and ZMPSTE24 (FACE-1) defects cause nuclear disorganization and identify restrictive dermopathy as a lethal neonatal laminopathy. Hum Mol Genet 13: 2493–2503. 15317753 10.1093/hmg/ddh265

[16] Oji V, Tadini G, Akiyama M, Blanchet BC, Bodemer C, et al (2010) Revised nomenclature and classification of inherited ichthyoses: results of the First Ichthyosis Consensus Conference in Soreze 2009. J Am Acad Dermatol 63: 607–641. 20643494 10.1016/j.jaad.2009.11.020

[17] Khnykin D, Miner JH, Jahnsen F (2011) Role of fatty acid transporters in epidermis: Implications for health and disease. Dermatoendocrinol 3: 53–61. 21695012 10.4161/derm.3.2.14816 PMC3117002

[18] Chen Q, Liang D, Overbeek PA (2008) Overexpression of E2F5/p130, but not E2F5 alone, can inhibit E2F-induced cell cycle entry in transgenic mice. Mol Vis 14: 602–614. 18385796 PMC2275213

[19] Moran JL, Bolton AD, Tran PV, Brown A, Dwyer ND, et al (2006) Utilization of a whole genome SNP panel for efficient genetic mapping in the mouse. Genome Res 16: 436–440. 16461637 10.1101/gr.4563306 PMC1415208

[20] Koster MI, Kim S, Mills AA, DeMayo FJ, Roop DR (2004) p63 is the molecular switch for initiation of an epithelial stratification program. Genes Dev 18: 126–131. 14729569 10.1101/gad.1165104 PMC324418

[21] Yuspa SH, Kilkenny AE, Steinert PM, Roop DR (1989) Expression of murine epidermal differentiation markers is tightly regulated by restricted extracellular calcium concentrations in vitro. J Cell Biol 109: 1207–1217. 2475508 10.1083/jcb.109.3.1207 PMC2115750

[22] Mills AA, Zheng B, Wang XJ, Vogel H, Roop DR, et al (1999) p63 is a p53 homologue required for limb and epidermal morphogenesis. Nature 398: 708–713. 10227293 10.1038/19531

[23] Jia Z, Moulson CL, Pei Z, Miner JH, Watkins PA (2007) Fatty Acid Transport Protein 4 Is the Principal Very Long Chain Fatty Acyl-CoA Synthetase in Skin Fibroblasts. J Biol Chem 282: 20573–20583. 17522045 10.1074/jbc.M700568200

[24] Lawson KA, Dunn NR, Roelen BA, Zeinstra LM, Davis AM, et al (1999) Bmp4 is required for the generation of primordial germ cells in the mouse embryo. Genes Dev 13: 424–436. 10049358 10.1101/gad.13.4.424 PMC316469

[25] Reneker LW, Chen Q, Bloch A, Xie L, Schuster G, et al (2004) Chick delta1-crystallin enhancer influences mouse alphaA-crystallin promoter activity in transgenic mice. Invest Ophthalmol Vis Sci 45: 4083–4090. 15505059 10.1167/iovs.03-1270

[26] Schneider C, Rasband W, Eliceiri E (2012) NIH Image to ImageJ: 25 years of image analysis. Nat Methods 9: 671–675. 22930834 10.1038/nmeth.2089 PMC5554542

[27] Umemoto H, Akiyama M, Yanagi T, Sakai K, Aoyama Y, et al (2011) New insight into genotype/phenotype correlations in ABCA12 mutations in harlequin ichthyosis. J Dermatol Sci 61: 136–139. 21168995 10.1016/j.jdermsci.2010.11.010

[28] Morice-Picard F, Leaute-Labreze C, Decor A, Boralevi F, LaCombe D, et al (2010) A novel mutation in the fatty acid transport protein 4 gene in a patient initially described as affected by self-healing congenital verruciform hyperkeratosis. Am J Med Genet A 152A: 2664–2665. 20815031 10.1002/ajmg.a.33648

[29] Khnykin D, Ronnevig J, Johnsson M, Sitek JC, Blaas HG, et al (2012) Ichthyosis prematurity syndrome: clinical evaluation of 17 families with a rare disorder of lipid metabolism. J Am Acad Dermatol 66: 606–616. 21856041 10.1016/j.jaad.2011.04.014

[30] Inhoff O, Hausser I, Schneider SW, Khnykin D, Jahnsen FL, et al (2011) Ichthyosis prematurity syndrome caused by a novel fatty acid transport protein 4 gene mutation in a German infant. Arch Dermatol 147: 750–752. 21690550 10.1001/archdermatol.2011.139

[31] Sobol M, Dahl N, Klar J (2011) FATP4 missense and nonsense mutations cause similar features in Ichthyosis Prematurity Syndrome. BMC Res Notes 4: 90. 21450060 10.1186/1756-0500-4-90 PMC3072334

[32] Paus R, Cotsarelis G (1999) The biology of hair follicles. N Engl J Med 341: 491–497. 10441606 10.1056/NEJM199908123410706

[33] Vielkind U, Hardy M (1996) Changing patterns of cell adhesion molecules during mouse pelage hair follicle development. 2. Follicle morphogenesis in the hair mutants, Tabby and downy. Acta Anat (Basel) 157: 183–194. 9226037 10.1159/000147880

[34] Davidson P, Hardy M (1952) The development of mouse vibrissae in vivo and in vitro. J Anat 86: 342–356. 12999638 PMC1273688

[35] Kulessa H, Turk G, Hogan BL (2000) Inhibition of Bmp signaling affects growth and differentiation in the anagen hair follicle. EMBO J 19: 6664–6674. 11118201 10.1093/emboj/19.24.6664 PMC305899

[36] Rendl M, Polak L, Fuchs E (2008) BMP signaling in dermal papilla cells is required for their hair follicle-inductive properties. Genes Dev 22: 543–557. 18281466 10.1101/gad.1614408 PMC2238674

[37] Byrne C, Tainsky M, Fuchs E (1994) Programming gene expression in developing epidermis. Development 120: 2369–2383. 7525178 10.1242/dev.120.9.2369

[38] Richard G, Bale SJ (2001) Autosomal Recessive Congenital Ichthyosis. In: Pagon RA, Bird TD, Dolan CR, et al.., editors. GeneReviews™. Seattle (WA): University of Washington, Seattle; 1993.

[39] Yanagi T, Akiyama M, Nishihara H, Sakai K, Nishie W, et al (2008) Harlequin ichthyosis model mouse reveals alveolar collapse and severe fetal skin barrier defects. Hum Mol Genet 17: 3075–3083. 18632686 10.1093/hmg/ddn204

[40] Huber M, Rettler I, Bernasconi K, Frenk E, Lavrijsen SP, et al (1995) Mutations of keratinocyte transglutaminase in lamellar ichthyosis. Science 267: 525–528. 7824952 10.1126/science.7824952

[41] Hopkin AS, Gordon W, Klein RH, Espitia F, Daily K, et al (2012) GRHL3/GET1 and Trithorax Group Members Collaborate to Activate the Epidermal Progenitor Differentiation Program. PLoS Genet 8: e1002829. 22829784 10.1371/journal.pgen.1002829 PMC3400561

[42] Ting SB, Caddy J, Wilanowski T, Auden A, Cunningham JM, et al (2005) The epidermis of grhl3-null mice displays altered lipid processing and cellular hyperproliferation. Organogenesis 2: 33–35. 19521564 10.4161/org.2.2.2167 PMC2634083

[43] Roca X, Olson AJ, Rao AR, Enerly E, Kristensen VN, et al (2008) Features of 5′-splice-site efficiency derived from disease-causing mutations and comparative genomics. Genome Res 18: 77–87. 18032726 10.1101/gr.6859308 PMC2134769

[44] Bhasi A, Pandey RV, Utharasamy SP, Senapathy P (2007) EuSplice: a unified resource for the analysis of splice signals and alternative splicing in eukaryotic genes. Bioinformatics 23: 1815–1823. 17344236 10.1093/bioinformatics/btm084

[45] Shapiro MB, Senapathy P (1987) RNA splice junctions of different classes of eukaryotes: sequence statistics and functional implications in gene expression. Nucleic Acids Res 15: 7155–7174. 3658675 10.1093/nar/15.17.7155 PMC306199

[46] Yusa K, Rashid ST, Strick-Marchand H, Varela I, Liu PQ, et al (2011) Targeted gene correction of alpha1-antitrypsin deficiency in induced pluripotent stem cells. Nature 478: 391–394. 21993621 10.1038/nature10424 PMC3198846

