# Correction: A Spontaneous *Fatp4/Scl27a4* Splice Site Mutation in a New Murine Model for Congenital Ichthyosis

**DOI:** 10.1371/journal.pone.0091624

**Published:** 2014-02-28

**Authors:** The PLOS ONE Staff

The following information was incorrectly omitted from the Funding section: "J.T. was in part supported by US National Institutes of Health grant R03AR061565."
